# Long-term effects of dietary protein and carbohydrate quality on prediabetes remission: results from the PREVIEW randomised multinational diabetes prevention trial

**DOI:** 10.1007/s00125-025-06560-x

**Published:** 2025-10-15

**Authors:** Ruixin Zhu, Jie Guo, Maija Huttunen-Lenz, Marta Silvestre, Gareth Stratton, Ian A. Macdonald, Teodora Handjieva-Darlenska, Svetoslav Handjiev, Santiago Navas-Carretero, Sally D. Poppitt, Mikael Fogelholm, Diego Martinez-Urbistondo, J. Alfredo Martinez, Anne Raben, Jennie Brand-Miller

**Affiliations:** 1 Key Laboratory of Precision Nutrition and Food Quality, Department of Nutrition and Health, China Agricultural University, Beijing, China; 2 Institute for Nursing Science, University of Education Schwäbisch Gmünd, Gmünd, Germany; 3 CINTESIS, NOVA Medical School, NOVA University of Lisbon, Lisbon, Portugal; 4 Applied Sports, Technology, Exercise and Medicine (A-STEM) Research Centre, Swansea University, Swansea, UK; 5 Division of Physiology, Pharmacology and Neuroscience, School of Life Sciences, Queen’s Medical Centre, Nottingham, UK; 6 Department of Pharmacology and Toxicology, Medical University of Sofia, Sofia, Bulgaria; 7 Centre for Nutrition Research, University of Navarra, Pamplona, Spain; 8 Centro de Investigacion Biomedica en Red Area de Fisiologia de la Obesidad y la Nutricion (CIBEROBN), Madrid, Spain; 9 IdisNA Instituto for Health Research, Pamplona, Spain; 10 Human Nutrition Unit, School of Biological Sciences, Department of Medicine, University of Auckland, Auckland, New Zealand; 11 Department of Food and Nutrition, University of Helsinki, Helsinki, Finland; 12 Internal Medicine Department, Clínica Universidad de Navarra, Madrid, Spain; 13 Precision Nutrition and Cardiometabolic Health Program, IMDEA Nutrition Institute (Madrid Institute for Advanced Studies), CEI UAM + CSIC, Madrid, Spain; 14 Department of Medicine and Endocrinology, University of Valladolid, Valladolid, Spain; 15 Department of Nutrition, Exercise and Sports, Faculty of Science, University of Copenhagen, Copenhagen, Denmark; 16 Department for Clinical and Translational Research, Copenhagen University Hospital – Steno Diabetes Center Copenhagen, Herlev, Denmark; 17 School of Life and Environmental Sciences and Charles Perkins Centre, University of Sydney, Sydney, Australia

**Keywords:** Diabetes prevention, Diet, Dietary composition, Glycaemic index, Glycaemic load, Normoglycaemia, Nutrition

## Abstract

**Aims/hypothesis:**

Recent studies advocate prediabetes remission as a goal in diabetes prevention, but the optimal dietary composition for prediabetes remission over the long term is unknown. We aimed to examine the long-term effects on prediabetes remission of a prudent diet with moderate protein and a moderate glycaemic index (GI) (akin to general dietary guidelines) vs a high-protein, low-GI diet.

**Methods:**

This study is a secondary analysis of PREVIEW, which is a 3 year, multicentre, parallel, randomised trial. Adults with overweight/obesity (BMI ≥ 25 and <30 kg/m^2^ or BMI ≥ 30 kg/m^2^, respectively) and prediabetes (fasting glucose 5.6–6.9 mmol/l and/or 2 h glucose 7.8–11.0 mmol/l determined using an OGTT) were recruited. Eligible participants underwent an 8-week rapid weight loss programme comprising a low-energy diet, followed by a 3 year weight maintenance phase comprising lifestyle intervention. At baseline, participants were randomly assigned to a high-protein (25% of energy from protein), low-GI (GI<50) diet, or a prudent diet with moderate protein (15% of energy from protein) and moderate GI (GI>56). The primary outcome of the current analysis was the number of participants who achieved prediabetes remission (i.e. a return to normal fasting glucose and normal glucose tolerance) at 1 year or 3 years. Secondary outcomes were changes in body weight and composition over 3 years (continuous variables) and maintenance of a ≥8% weight loss target (binary variable). Modified intention-to-treat analyses were performed on all participants who received the dietary intervention (*n*=1856). Risk ratios and 95% CI for prediabetes remission and maintaining the weight loss target in each diet group were estimated using multilevel modified Poisson regression adjusted for age and sex. Linear mixed models were used to estimate the dietary effects on changes in body weight and composition.

**Results:**

The moderate-protein, moderate-GI group (*n*=923) had a higher rate of remission than the high-protein, low-GI group (*n*=933) at both 1 year (rate of remission 26.3% vs 20.7%; RR 1.26; 95% CI 1.04, 1.53; *p*=0.025) and 3 years (20.6% vs 15.5%; RR 1.26; 95% CI 1.06, 1.50; *p*=0.015). However, body weight and composition changes were similar for participants on the moderate-protein, moderate-GI vs high-protein, low-GI diet at 1 year (54.0% vs 57.3% of participants met the weight loss maintenance target [≥8% of initial body weight]; *p*=0.215) and 3 years (31.4% vs 30.4%, respectively; *p*=0.793). The differences in remission rates of the two dietary patterns were independent of body weight and composition changes.

**Conclusions/interpretation:**

Following rapid weight loss, a prudent diet with moderate protein and moderate GI was more effective for long-term prediabetes remission than a high-protein, low-GI diet, irrespective of weight change.

**Trial registration:**

ClinicalTrials.gov NCT01777893

**Graphical Abstract:**

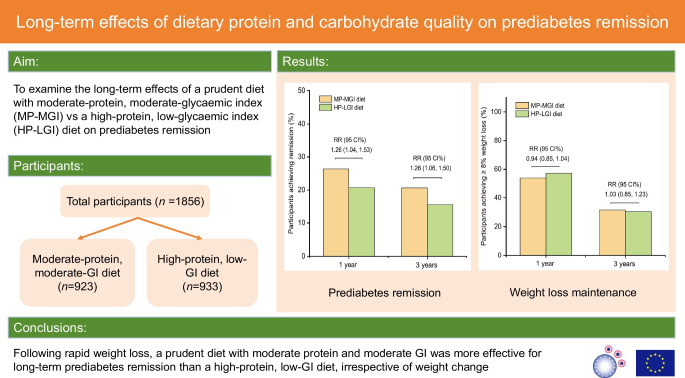

**Supplementary Information:**

The online version of this article (10.1007/s00125-025-06560-x) contains peer-reviewed but unedited supplementary material.


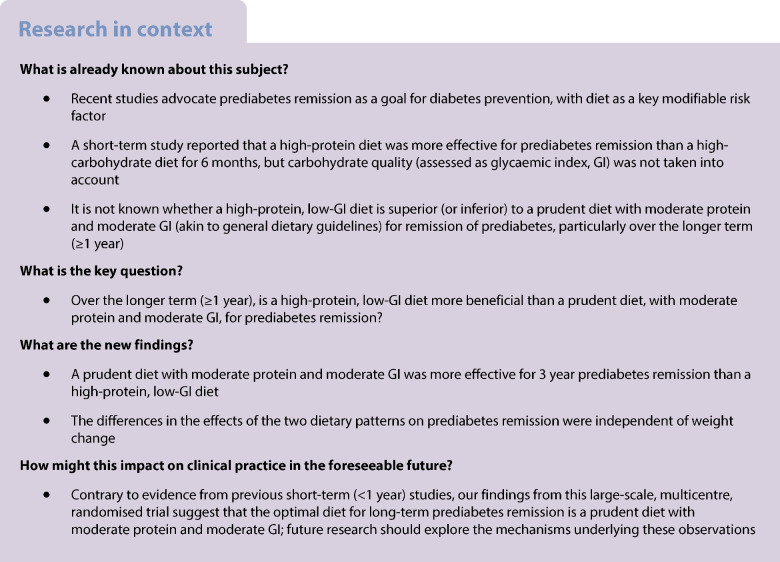


## Introduction

By 2050, diabetes is expected to affect 1.3 billion people worldwide, with the vast majority having type 2 diabetes [1]. Prevention of type 2 diabetes will not only avert complications, but also produce enormous savings to healthcare budgets [2]. Prediabetes is the strongest risk factor for diabetes [3]. Prediabetes remission, a return to normal glucose regulation, is related to lower risks of diabetes [4–6], complications [7] and mortality [8]. Prediabetes remission is therefore advocated for use as a goal in diabetes prevention [4, 5]. Obesity is highly prevalent among people with prediabetes, and there is strong evidence that weight loss is effective for diabetes prevention [9] and prediabetes remission [10]. The ADA recommends a weight loss target of ≥7% for diabetes prevention [9]. Unfortunately, weight regain after weight loss is common [8], and it is unclear to what extent this compromises prediabetes remission.

Despite recent advances in drug therapy, dietary strategies are still first-line and effective treatment for weight management and diabetes prevention [11]. Identifying the optimal dietary pattern for prediabetes remission is important for diabetes prevention. Low-carbohydrate diets are effective for weight loss and glucose management in the short term (<1 year) [12–14]. To achieve long-term (≥1 year) benefits, some successful diabetes prevention studies involving lifestyle intervention, such as the US Diabetes Prevention Program [15] and the Finnish Diabetes Prevention Study [16], were based on a prudent, higher-carbohydrate diet (45-65% of energy [E%], akin to general dietary guidelines [17]) with moderate protein (10–20 E%) and moderate glycaemic index (GI). A recent study also showed that a high-protein diet (30 E%) with increased intake of low-fat animal foods was more effective for prediabetes remission than a high-carbohydrate diet (15 E% as protein) for 6 months [18, 19]. As well as carbohydrate quantity, recent meta-analyses have demonstrated that carbohydrate quality (GI and glycaemic load) may also play a role in weight and glucose management [20]. In particular, the DiOGenes study reported that a high-protein (23–28 E%, with intake of fish twice a week), low-GI diet was more effective for weight maintenance for 6 months than four other diets that varied in composition with respect to both protein and carbohydrate quality [21]. However, the long-term (≥1 year) effects of dietary composition (especially protein content and carbohydrate quality) on prediabetes remission remain unknown.

This study is a secondary analysis of PREVIEW, a 3 year, multinational, randomised diabetes prevention trial. This analysis aimed to examine the effects of a high-protein, low-GI diet vs a prudent diet with moderate protein and moderate GI on prediabetes remission (i.e. return to normoglycaemia, similar to the ADA definition of complete diabetes remission [22]) and maintenance of the weight loss target at 1 and 3 years. We also examined whether the effects varied between adults who achieved and did not achieve the weight maintenance target, as well as exploring the role of changes in body weight and composition in prediabetes remission.

## Methods

### Study design

This secondary analysis used the data from the PREVIEW study (Prevention of Diabetes through Lifestyle Intervention and Population Studies in Europe and Around the World) (ClinicalTrials.gov: NCT01777893), a 3 year, multicentre, open-label, parallel, RCT conducted between June 2013 and March 2018 at eight intervention centres in Denmark, Finland, the UK, the Netherlands, Spain, Bulgaria, Australia and New Zealand. The main aim of the PREVIEW study was to examine the effects of two diets on prevention of type 2 diabetes among adults with overweight/obesity and prediabetes. Participants underwent an 8-week rapid weight loss programme comprising a low-energy diet (phase 1), followed by a 148-week weight loss maintenance phase (phase 2) comprising lifestyle intervention (diet and physical activity, PA). In phase 1, all eligible participants were provided with low-energy diet meal replacement products with an intake of 3400 kJ/day (810 kcal/day). Those who lost ≥8% of their initial body weight after phase 1 were allowed to enter phase 2 and underwent lifestyle intervention.

The primary outcome of the PREVIEW study was the difference in 3 year cumulative incidence of type 2 diabetes between the two diets (high-protein, low-GI vs moderate-protein, moderate-GI). Detailed information on the study design and findings is provided in the PREVIEW protocol and the main paper [23, 24]. The protocol was approved by the human ethics committee at each intervention centre (see electronic supplementary material [ESM] Methods). The original ethics approvals allowed for secondary analyses based on the database created during the PREVIEW study and also for subgroup studies at each site, which were subject to separate ethics approvals. The PREVIEW study was conducted in line with the Declaration of Helsinki.

### Study population and randomisation

Community-based participants were enrolled between June 2013 and April 2015. Eligible participants were women and men with a BMI ≥25 kg/m^2^ and prediabetes. The participants were not recruited through random sampling. However, during recruitment, we paid special attention to sex and age distribution. We aimed to avoid significant sex imbalance and to achieve a wide range of ages (25–70 years) to increase generalisability. Sex was self-reported by participants as female or male. Ethnicity/race was self-reported by participants as white, Asian, Black, Arabic, Hispanic or other. Prediabetes was defined as fasting glucose of 5.6–6.9 mmol/l and/or 2 h glucose of 7.8–11.0 mmol/l determined using an OGTT, according to ADA criteria. The main exclusion criteria were pre-existing type 1 or 2 diabetes. Inclusion and exclusion criteria and reasons for attrition during the study have been listed elsewhere [23, 24]. Written informed consent was provided by eligible participants.

At baseline, participants were randomly assigned in a 1:1 ratio to one of the two diet groups, and those in each diet group were randomised (1:1) into one of two PA groups (moderate vs high intensity). The randomisation was stratified by sex (female/male) and age group (25–45, 46–54 and 55–70 years) at each centre. Detailed information is provided in ESM Methods.

### Interventions

At the start of the weight loss maintenance phase, participants received instructions for one of the two diets and one of the two PA programmes (four intervention groups in total). Detailed group information is provided in ESM Methods. The two diets had different macronutrient composition and carbohydrate quality: a high-protein (25 E%), lower-carbohydrate (45 E%), low-GI (<50) diet, or a moderate-protein (15 E%), higher-carbohydrate (55 E%), moderate-GI (>56) diet. Details about GI levels are provided in ESM Methods. Based on previous research (i.e. the DiOGenes study) [21], we anticipated a difference of approximately 5 units in the mean GI values between the two diet groups was necessary to detect a difference in 3 year incidence of type 2 diabetes.

Diets were consumed ad libitum without energy intake targets. Participants received diet and PA advice from trained professionals. Both intervention diets emphasised healthy food choices, and participants in each diet group were encouraged to increase intakes of wholegrain cereals, vegetables and fruit, and reduce intakes of red meat and sugar-sweetened beverages. Participants in the high-protein, low-GI group were recommended to eat more low-GI cereals, pasta, legumes, low-fat dairy products, poultry and fish. Participants in the moderate-protein, moderate-GI group were recommended to eat more wholegrain cereals with moderate GI. Details of PA programmes are provided in ESM Methods. Participants were encouraged to maintain target weight loss during the weight loss maintenance phase.

Dietary intake and PA were assessed using self-reported 4-day food records and 7-day accelerometry, respectively. Details about diet and PA assessment are provided in ESM Methods, including standard operating procedures and compliance strategies.

### Outcomes

The primary outcome of the current analysis was the number of participants who achieved both normal fasting glucose and normal glucose tolerance at 1 year and 3 years (with normal fasting glucose and normal glucose tolerance at 3 years indicating complete prediabetes remission, corresponding to the ADA definition of diabetes remission [22]). Prediabetes was defined as elevated fasting plasma glucose (5.6‒6.9 mmol/l) and 2 h plasma glucose (7.8‒11.0 mmol/l) after an OGTT. Prediabetes remission was defined as the return from prediabetes criteria to normal measures of glucose metabolism (normoglycaemia) at 1 and 3 years. Normoglycaemia was defined as normal fasting glucose (<5.6 mmol/l) and normal glucose tolerance (2 h glucose <7.8 mmol/l) [4, 5]. In the sensitivity analyses, prediabetes remission is defined as meeting all of the following three criteria: a return to normal HbA_1c_ (<39 mmol/mol or 5.7%), normal fasting glucose (<5.6 mmol/l), and normal 2 h glucose (<7.8 mmol/l) at 1 and 3 years. We used a derived variable conditioned on all three criteria for analysis.

The secondary outcomes of interest were: (1) count variables: the number of participants achieving the PREVIEW weight loss target (i.e. ≥8% of initial body weight after rapid weight loss phase according to protocol) at 1 and 3 years; the ADA weight loss target (i.e. ≥7% of initial body weight) was used in the sensitivity analysis; (2) continuous variables: changes in body weight, BMI, fat mass and fat-free mass over 3 years as measured by absolute value at baseline, 8 weeks, 1 year and 3 years; and (3) continuous variables: changes in surrogate adiposity markers, including fat-free mass index, fat mass index, WHR, a body shape index (ABSI), visceral adiposity index (VAI) and lipid accumulation product (LAP) over 3 years (absolute measured values rather than changes in variables at baseline, 8 weeks, 1 year and 3 years). The formulas used to calculate ABSI, VAI and LAP are listed in ESM Methods. Outcomes were measured according to pre-specified standard operating procedures. The assessment methods used have been described previously [24] and in ESM Methods.

### Statistical analysis

We conducted modified intention-to-treat analyses and included the participants who achieved ≥8% weight loss at the end of the weight loss phase and received the lifestyle intervention for weight maintenance. The RR and 95% CI for prediabetes remission and achieving the weight loss target at 1 and 3 years between the two diet groups were estimated using multilevel modified Poisson regression, adjusting for sex and age group as fixed effects and intervention centre as a random effect. We further compared the differences in body weight and composition measures, insulin resistance and beta cell function between the two diet groups and between participants with and without remission within each diet group. The role of the change in body weight and composition measures in the effects of the two diets on the rate of prediabetes remission was examined by additionally adjusting for change in body weight and composition measures in the models. The interaction of diets and maintenance of the weight loss target was examined by additionally adding an interaction term. For changes in body weight and composition over 3 years, linear mixed-effect models were used to manage the repeated measurements of body weight and composition at baseline, 8 weeks, 1 year and 3 years. Changes in body weight and composition measures between the two diet groups over 3 years were estimated using linear mixed models, adjusting for sex and age group as fixed effects and intervention centre as a random effect. Missing data were not imputed.

For the primary outcome of the current analysis (i.e. prediabetes remission at 1 and 3 years), we performed several sensitivity analyses and auxiliary analyses. Details are provided in ESM Methods. For the primary outcome (i.e. prediabetes remission at 1 and 3 years), significance level α values were adjusted for multiple comparisons. For other outcomes, adjustments for multiple comparisons were not employed, and related results should be interpreted as exploratory due to the potential for type I error. Data were analysed using SAS software version 9.4 (SAS Institute, Cary, NC) and SPSS software version 28.0 (IBM, Chicago, IL). The statistical test was two-sided. A *p* value ≤ 0.025 was considered statistically significant for the primary outcome. A *p* value ≤ 0.05 was considered statistically significant for other outcomes.

## Results

### Participants

This secondary analysis included 1856 participants (ESM Fig. 1). Of these, 933 were in the high-protein, low-GI group, and 923 were in the moderate-protein, moderate-GI group. In total, 475 (26%) participants had dropped out by 1 year and 894 (48%) had dropped out by 3 years. The baseline characteristics of participants were broadly similar between the diet groups (Table 1). Female participants represented 66% of the participants. Compared with completers, non-completers were younger and heavier (ESM Table 1).

**Table 1:** Participant characteristics at baseline

	HP-LGI group (*N*=933)	MP-MGI group (*N*=923)
Age range, years	25–70	25–70
Age, years	55 (43–62)	55 (43–62)
Age group, years		
25–45	302 (32.4)	287 (31.1)
46–54	143 (15.3)	148 (16.0)
55–70	488 (52.3)	488 (52.9)
Sex		
Female	619 (66.3)	614 (66.5)
Male	314 (33.7)	309 (33.5)
Race and ethnicity		
White	843 (90.4)	820 (88.8)
Other^a^	90 (9.6)	103 (11.2)
Body weight, kg	96.8 (84.7–110.5)	96.2 (84.7–109.7)
Height, m	1.67 (1.61–1.75)	1.67 (1.62–1.75)
BMI, kg/m^2^	33.9 (30.7–38.1)	33.9 (30.8–38.2)
Weight status		
Overweight^b^	173 (18.5)	185 (20.0)
Obesity^c^	760 (81.5)	738 (80.0)
Fat mass, kg	40.3 (33.3–50.0)	40.9 (33.4–49.7)
Fat mass index, kg/m^2^	14.4 (11.7–17.9)	14.5 (11.9–18.0)
Fat-free mass, kg	54.0 (47.8–64.1)	53.8 (47.6–64.2)
Fat-free mass index, kg/m^2^	19.6 (18.0–21.5)	19.3 (17.8–21.3)
Waist circumference, cm	109.3 (101.0–118.7)	109.0 (99.6–118.4)
Hip circumference, cm	115.5 (108.5–125.5)	115.8 (108.8–124.8)
WHR	0.93 (0.87–1.00)	0.93 (0.87–1.00)
ABSI	0.080 (0.076–0.084)	0.080 (0.076–0.084)
VAI	1.87 (1.29–2.76)	1.82 (1.31–2.65)
LAP	65.0 (45.1–96.0)	64.0 (44.4–93.9)
Fasting plasma glucose, mmol/l	6.2±0.7	6.2±0.7
2 h plasma glucose, mmol/l	7.7±2.2	7.6±2.1
HbA_1c_, mmol/mol	36.6±3.7	36.7±4.0
HbA_1c_, %	5.5±0.3	5.5±0.4

Values are mean±SD or median (25th–75th percentiles) for continuous variables and *n* (%) for categorical variables

^a^Including Asian, Black, Arabic, Hispanic and other

^b^Overweight is defined as a BMI ≥25 and <30 kg/m^2^

^c^Obesity is defined as a BMI ≥30 kg/m^2^

HP-LGI, high-protein, low-GI diet; MP-MGI, moderate-protein, moderate-GI diet

### Dietary composition

ESM Tables 2 and 3 show that the high-protein, low-GI and moderate-protein, moderate-GI groups had similar baseline dietary intake and PA. As planned, the moderate-protein, moderate-GI group had significantly lower protein intake, higher GI and a higher carbohydrate intake than the high-protein, low-GI group at 1 and 3 years (at 1 year: mean values of GI 54.6 [SE 0.4] vs 51.0 [SE 0.4]; mean values of protein 18.4 E% [SE 0.2] vs 22.1 E% [SE 0.2]; at 3 years: mean values of GI 54.3 [SE 0.4] vs 51.6 [SE 0.5]; mean values of protein 18.8 E% [SE 0.3] vs 21.2 E% [SE 0.3]). There were no significant differences in fat, fibre or energy intake between the diet groups (*p* for group and time interaction >0.05 for all; *p* for group >0.05 for all). There were no differences in energy intake between the PA groups within each dietary arm.

### Prediabetes remission

**Fig. 1:**
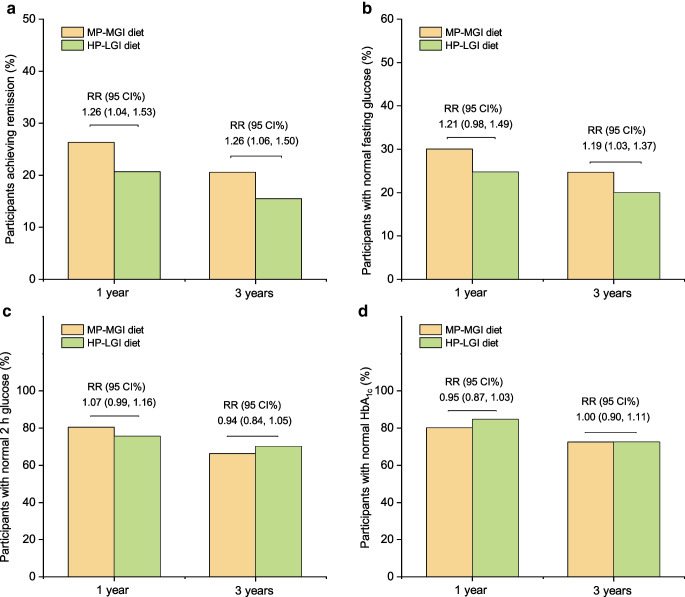
Effects of dietary patterns on prediabetes remission and glucose management over 3 years. (**a**) Proportion of participants who achieved prediabetes remission in each diet group. (**b**) Proportion of participants with normal fasting glucose in each diet group. (**c**) Proportion of participants with normal 2 h glucose in each diet group. (**d**) Proportion of participants with normal HbA_1c_ in each diet group. Prediabetes remission was defined as normal fasting glucose and normal 2 h fasting glucose. The high-protein, low-GI diet was the reference group. The RR and 95% CI at 1 and 3 years between the diet groups were estimated using multilevel modified Poisson regression models, adjusting for sex (male/female) and age group (25–45, 46–54 and 55–70 years of age) as fixed effects and intervention centre as a random effect. The data analyses were based on modified intention-to-treat analyses without imputation for missing data. HP-LGI, high-protein, low-GI diet; MP-MGI, moderate-protein, moderate-GI diet

In the modified intention-to-treat analyses, the moderate-protein, moderate-GI group had a higher rate of prediabetes remission (defined using fasting glucose and 2 h glucose) at 1 and 3 years than the high-protein, low-GI group (rate of remission at 1 year 26.3% [159 of 604] vs 20.7% [134 of 646]; RR 1.26; 95% CI 1.04, 1.53; *p*=0.025; rate of remission at 3 years 20.6% [97 of 472] vs 15.5% [73 of 471]; RR 1.26; 95% CI 1.06, 1.50; *p*=0.015) (Fig. 1a). The above results were robust in sensitivity analyses in which prediabetes remission was defined using alternative criteria including HbA_1c_, in which missing data were imputed, and in which the PA group was further adjusted for (ESM Figs 2–4). After adjusting for energy intake, the results remained robust at 1 year (ESM Fig. 5). At 3 years, similar trends were observed, although the results were not significant due to a high number of missing values for energy intake and the smaller sample size (ESM Fig. 5). In the complete-case analysis, the results remained robust at 3 years (ESM Fig. 6). Similar trends were observed at 1 year (a 4.1% increase in the rate of prediabetes remission in the moderate-protein, moderate-GI group), although these were not significant due to the smaller sample size (ESM Fig. 6).

At baseline, there were no differences in the proportion of participants with normal fasting glucose, normal 2 h glucose or normal HbA_1c_ between the two diet groups (*p*<0.05 for all). The moderate-protein diet resulted in significantly more participants achieving normal fasting glucose at 3 years (24.7% vs 20.0%; RR 1.19; 95% CI 1.03, 1.37; *p*=0.026), but the two diets did not differ in terms of the other glucose criteria (Fig. 1b–d). The results of the auxiliary analyses are shown in ESM Results.

### Body weight and composition

**Fig. 2:**
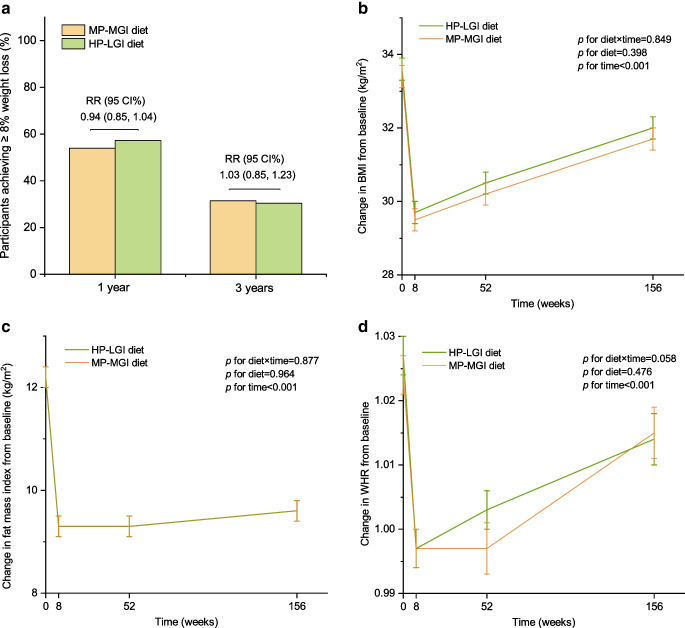
Effects of dietary patterns on maintenance of weight loss target and changes in body composition over 3 years. (**a**) Proportion of participants who achieved the weight loss target (≥8% of initial body weight) in each diet group. The high-protein, low-GI diet was the reference group. (**b**) Change in BMI over 3 years (estimated marginal mean and SE). (**c**) Change in fat mass index over 3 years (estimated marginal mean and SE). (**d**) Change in WHR over 3 years (estimated marginal mean and SE). RR and 95% CI for maintenance of weight loss targets at 1 and 3 years between the diet groups were estimated using multilevel modified Poisson regression models, adjusting for sex (male/female) and age group (25–45, 46–54 and 55–70 years of age) as fixed effects and intervention centre as a random effect. Changes in BMI, fat mass index and WHR over 3 years between the diet groups were estimated using linear mixed models, adjusting for sex and age group as fixed effects and intervention centre as a random effect. HP-LGI, high-protein, low-GI diet; MP-MGI, moderate-protein, moderate-GI diet

In the modified intention-to-treat analyses, 54.0% (352 of 652) of participants in the moderate-protein, moderate-GI group maintained the weight loss target (≥8% of body weight) at 1 year, compared with 57.3% (352 of 614) of those in the high-protein, low-GI group. At 3 years, 31.4% (154 of 490) and 30.4% (151 of 497), respectively, maintained the weight loss target (Fig. 2a). There were no differences in the proportion of participants who achieved weight loss targets between the diet groups at 1 year (RR 0.94; 95% CI 0.85, 1.04; *p*=0.215) and 3 years (RR 1.03; 95% CI 0.85, 1.23; *p*=0.793). The above results were robust in the sensitivity analysis in which the weight loss target was defined according to the ADA recommendation, i.e. ≥7% of initial body weight (ESM Fig. 7). There were no differences between the diet groups when outcomes were expressed as changes in body weight or body composition, including BMI, fat mass, fat mass index, fat-free mass, fat-free mass index, WHR, ABSI and VAI, over 3 years (Fig. 2b–d and ESM Table 4).

### Role of body weight and composition in prediabetes remission

The changes in body weight and composition from baseline to 1 or 3 years were similar across diet groups (ESM Table 5). Greater reductions in body weight, BMI and fat mass were observed among those who achieved remission and those who did not within each diet group (ESM Table 6). After adjusting for maintenance of the weight loss target (≥8% or ≥7%) or changes in body weight and composition from baseline (Table 2), the effect sizes (RR) for prediabetes remission between the diet groups at 1 and 3 years remained similar, and *p* values remained significant in most cases. There was no significant interaction between the diet groups and maintenance of ≥8% or ≥7% weight loss at 1 year (*p* for interaction >0.05). However, at 3 years, the effects of the moderate-protein, moderate-GI diet on remission of prediabetes were stronger in individuals who maintained ≥8% or ≥7% weight loss (*p* for interaction <0.05) (ESM Results and ESM Table 7).

**Table 2:** Role of body weight and composition measures in the effects of dietary patterns on prediabetes remission

	Prediabetes remission at 1 year			Prediabetes remission at 3 years		
	HP-LGI	MP-MGI	*p* value	HP-LGI	MP-MGI	*p* value
Model 1	1 (reference)	1.26 (1.04, 1.53)	0.025	1 (reference)	1.26 (1.06, 1.50)	0.015
Model 1 + additional adjustment for the following measures						
Maintenance of weight loss targets (categorical)	1 (reference)	1.26 (1.04, 1.53)	0.027	1 (reference)	1.25 (1.06, 1.48)	0.016
Percentage weight loss	1 (reference)	1.21 (1.00, 1.48)	0.053	1 (reference)	1.20 (1.01, 1.43)	0.044
Change in body weight from baseline	1 (reference)	1.23 (1.01, 1.51)	0.043	1 (reference)	1.22 (1.03, 1.44)	0.027
Change in BMI from baseline	1 (reference)	1.24 (1.01, 1.51)	0.039	1 (reference)	1.21 (1.02, 1.44)	0.034
Change in fat mass from baseline	1 (reference)	1.23 (1.02, 1.50)	0.038	1 (reference)	1.24 (1.05, 1.47)	0.020
Change in fat mass index from baseline	1 (reference)	1.24 (1.02, 1.50)	0.035	1 (reference)	1.23 (1.04, 1.47)	0.025
Change in fat-free mass from baseline	1 (reference)	1.26 (1.04, 1.54)	0.026	1 (reference)	1.29 (1.09, 1.54)	0.010
Change in fat-free mass index from baseline	1 (reference)	1.26 (1.04, 1.54)	0.026	1 (reference)	1.29 (1.09, 1.54)	0.010
Change in WHR from baseline	1 (reference)	1.26 (1.04, 1.52)	0.025	1 (reference)	1.28 (1.07, 1.54)	0.014
Change in ABSI from baseline	1 (reference)	1.26 (1.04, 1.52)	0.023	1 (reference)	1.27 (1.06, 1.52)	0.018
Change in VAI from baseline	1 (reference)	1.27 (1.03, 1.57)	0.030	1 (reference)	1.26 (1.07, 1.49)	0.013
Change in LAP from baseline	1 (reference)	1.28 (1.04, 1.57)	0.026	1 (reference)	1.28 (1.08, 1.51)	0.010

Values are RR (95% CI)

Analyses were performed using modified Poisson regression models. Model 1 was adjusted for sex (male/female) and age group (25–45, 46–54 and 55–70 years of age) as fixed effects and intervention centre as a random effect. Other models were additionally adjusted for changes in body weight and composition measures from baseline to 1 and 3 years

HP-LGI, high-protein, low-GI diet; MP-MGI, moderate-protein, moderate-GI diet

## Discussion

This secondary analysis of the PREVIEW multinational randomised diabetes prevention trial provides novel, high-quality evidence on the role of dietary composition per se in long-term prediabetes remission. We found that a high-protein, lower-carbohydrate, low-GI diet was less effective for long-term prediabetes remission compared with a prudent, higher-carbohydrate diet (as in general dietary guidelines) with moderate protein and moderate GI. These results were robust in sensitivity analyses. Surprisingly, the differences in prediabetes remission rates were independent of changes in body weight and body composition.

Previous clinical trials have explored the effects of dietary composition on glucose management, but few have compared them for as long as 3 years. Low-carbohydrate or low-energy diets have been found to be effective for weight loss and glucose management in the short term, but the majority of studies only lasted 2–6 months, and little is known about the long-term effects of such diets [12–14]. Moreover, weight regain is common after rapid weight loss [25, 26], and therefore it is necessary to identify an ideal and sustainable diet for long-term weight loss maintenance and glucose management. In terms of long-term effects, low-carbohydrate diets may not be that effective in some populations. Indeed, a recent prospective cohort study showed that low-carbohydrate diets were related to increased diabetes risks in an Australian population [27]. However, the importance of carbohydrate quality and the benefits of low-GI diets have been confirmed by meta-analyses of randomised trials [20] and large prospective cohorts [28].

In the present study, in which total energy intake was not restricted, the moderate-GI diet (with higher carbohydrate) outperformed the lower-carbohydrate, lower-GI diet in terms of prediabetes remission, but the diets were comparable with respect to diabetes incidence and other markers of glycaemic control. This unexpected result may be partly attributable to both diet groups adhering to healthy eating in the PREVIEW study. All participants were given lifestyle instruction from trained professionals, e.g. on sufficient dietary fibre and low intake of added sugars. According to our analysis, the intake of dietary fibre was comparable in both groups throughout the intervention. While a review of meta-analyses concluded that fibre intake was more important than carbohydrate amount for people with diabetes [29], our findings suggest that carbohydrate may actually be beneficial as long as fibre intake is high enough (>20 g) and overall diet quality is high. Moreover, unlike other large diabetes prevention trials, the large initial weight loss induced rapidly by a low-energy formula diet resulted in prediabetes remission in most participants.

Some previous studies have supported the use of high-protein diets for glucose management [13], potentially because they increase secretion of glucagon-like peptide 1 [30]. In contrast to the present study, a small, short-term randomised trial (*n*=24) demonstrated that participants in the high-protein group had higher prediabetes remission than those in the high-carbohydrate group, even with similar weight loss over 6 months [18, 19]. Nonetheless, our findings and a recent meta-analysis of randomised trials do not support the use of higher protein diets for prediabetes remission or lowering HbA_1c_ or fasting plasma glucose [31]. Similarly, a recent umbrella review of systematic reviews of prospective cohort studies suggested that higher total protein intake was associated with a higher diabetes risk, with no strong evidence for or against animal vs plant protein [31]. Future studies should assess the long-term effects of high-plant vs high-animal protein on prediabetes remission.

Intake of dietary protein is generally considered to be beneficial to weight management, through mechanisms that are hypothesised to include increased satiety hormones [32]. However, in the PREVIEW study, the weight loss maintenance and changes in body composition were similar on the two diets, despite a large difference between groups in terms of target protein intake (25 vs 15 E%) and recorded protein intake (22 vs 18 E%). Previous studies have found that a combination of high protein and low-GI carbohydrates could help with weight loss and appetite control. This pattern was the optimal diet for weight maintenance in the DiOGenes study, but the dietary intervention lasted only 26 weeks [21, 33]. In the PREVIEW study, the high-protein, low-GI diet suppressed self-reported hunger more effectively than the moderate-protein, moderate-GI diet [34]. However, it was not superior in terms of weight loss maintenance or for improvements in body composition. Both diet groups were given healthy diet advice with increases in wholegrain cereals, vegetables and fruits, and decreases in red meat and added sugar. Similarly, an umbrella review of 19 meta-analyses of hypocaloric diets for weight management in people with type 2 diabetes found no support for any particular macronutrient profile over another [35]. Branched-chain amino acids (i.e. leucine, isoleucine and valine) have been suggested to have adverse effects on longevity and insulin resistance [36]. The effects are not consistent, however, and may depend on the background diet and its fat content. Future studies should therefore endeavour to separate the effects of high-protein diets from those high in branched-chain amino acids as well as assessing plant vs animal protein.

It is worth noting that approximately 66% of PREVIEW participants were women and approximately 70% of them were middle-aged or older. Unlike men, the basal metabolic rate of women decreases significantly during midlife due to changes in sex steroid hormones as part of menopause [37]. Weight gain and metabolic disorders are common among this age group [37]. Our study provides reassurance that a prudent diet containing moderate (rather than high) levels of protein and higher levels of carbohydrate is effective for prediabetes remission among perimenopausal and postmenopausal women with a high-protein, lower-carbohydrate diet.

This secondary analysis has many strengths, including the multinational randomised trial design, long duration of intervention, large sample size, broad age range, use of a centralised laboratory, repeated measurements, sensitivity analyses and consistent findings from multiple analyses. Certain limitations merit consideration. First, all diet studies introduce bias because participants are aware of which diet they have been allocated and their food records may not be accurate. According to the food records, the dietary intervention did not fully achieve the macronutrient targets. The differences in carbohydrate intake (approximately 6 E%), protein intake (approximately 3 E%) and GI (approximately 3 units) between the two diets were smaller than predicted. In particular, the GI value (54) in the moderate-GI group was lower than targeted (>56). Second, there were no differences between groups in terms of whether recorded PA was moderate or high intensity, regardless of which group they had been allocated to, which made us unable to examine the effect of the potential interaction between PA and diet on prediabetes remission. Third, this analysis was specified in the PREVIEW synopsis for publication, but not in the PREVIEW study protocol. Fourth, the attrition rate was acceptable at 1 year (26%), but was higher than expected (48%) at the end of the study, resulting in selection bias. When we imputed missing data to reduce bias, our results remained robust in multiple sensitivity analyses, especially complete-case analyses among older and healthier participants. Fifth, we have no knowledge of the prior duration of prediabetes in our participants. As the participants were randomly assigned, the baseline duration of prediabetes should be balanced and not affect the results. However, the rate of achieving prediabetes remission may be affected by prediabetes duration, which may lead to differences in incidence in the results of this study compared with others. Finally, the PREVIEW participants were predominantly of Northern European origin (approximately 90%), which limits the generalisability of our findings to other ethnicities. This study has not been externally validated, particularly in participants of other ethnicities.

In conclusion, this secondary analysis provides new insights into the role of dietary composition in long-term prediabetes remission. Our findings indicate that a prudent, higher-carbohydrate diet with moderate protein and moderate GI (similar to general dietary guidelines around the world) was more effective for long-term prediabetes remission than a high-protein, lower-carbohydrate, lower-GI diet, regardless of weight change, in a predominantly white older population, two-thirds of whom were women. These findings suggest caution be used in recommending high-protein diets in this context. However, given the limitations of our study (e.g. the lack of external validation), these findings should be confirmed by future trials, and the underlying mechanisms warrant further investigation.

## Supplementary Information

Below is the link to the electronic supplementary material.

ESM (PDF 1775 KB)

## Data Availability

The datasets analysed during the current study are available from the corresponding author on reasonable request.

## Abbreviations

ABSI: A body shape index
E%: Percentage of energy derived from each macronutrient
GI: Glycaemic index
LAP: Lipid accumulation product
PA: Physical activity
VAI: Visceral adiposity index

## Authors’ relationships and activities

IAM was a member of the UK Government Scientific Advisory Committee on Nutrition, the Mars Scientific Advisory Council, the Mars Europe Nutrition Advisory Board, the Nestlé Research Scientific Advisory Board and the Novozymes Scientific Advisory Board, and was a Scientific Advisor to the Waltham Centre for Pet Nutrition until 2020. On 1 August 2020, he became Professor Emeritus at the University of Nottingham and took up the post of Scientific Director of the Nestlé Institute of Health Sciences in Lausanne, Switzerland, which terminated in August 2022. SDP was the Fonterra Chair in Human Nutrition during the PREVIEW intervention. AR has received honoraria from the International Sweeteners Association, Nestlé and Unilever, and research funds from Novo Nordisk A/S. JB-M reports a relationship with the China National Research Institute of Food and Fermentation Industries that includes consulting or advisory roles, speaking and lecture fees, and travel reimbursement. She also reports a relationship with the Novo Nordisk Foundation that includes consulting or advisory roles, and a relationship with Hachette Livre that includes being a co-author of books about the glycaemic index of foods and diabetes. She oversees a glycaemic index testing service at the University of Sydney. She was President of the Glycemic Index Foundation between 2002 and 2024. She is on the editorial boards of the *American Journal of Clinical Nutrition* and the *International Journal of Obesity* and the scientific board of Zoe Global, a personalised nutrition company. The remaining authors declare that there are no relationships or activities that might bias, or be perceived to bias, their work.
