# Supplementary material for: Long-term effects of dietary protein and carbohydrate quality on prediabetes remission: results from the PREVIEW randomised multinational diabetes prevention trial
Source: Diabetologia. 2025 Oct 15;69(1):81–92. doi: 10.1007/s00125-025-06560-x (PMC12686045; doi:10.1007/s00125-025-06560-x)
Supplement: Supplementary file 1 — ESM (PDF 1775 KB) [file 125_2025_6560_MOESM1_ESM.pdf]

Electronic supplementary material

Long-term effects of dietary protein and carbohydrate quality on prediabetes remission: results from the PREVIEW randomised multinational diabetes prevention trial

ESM Methods

Study design

The PREVIEW protocol was approved by the following Human Ethics Committee: the Research Ethics Committees of the Capital Region (University of Copenhagen, Denmark), Coordinating Ethical Committee of HUS (Helsinki and Uusimaa Hospital District) (University of Helsinki, Finland), Medical Ethics Committee of the Maastricht University Medical Centre (University of Maastricht, the Netherlands), UK National Research Ethics Service (NRES) and East Midlands (Leicester) Ethics Committee (University of Nottingham, the UK), Research Ethics Committee of the University of Navarra (University of Navarra, Spain), Commission on Ethics in Scientific Research with the Medical University-Sofia (KENIMUS) (Medical University of Sofia, Bulgaria), The University of Sydney, Human Research Ethics Committee (HREC) (University of Sydney, Australia), Health and Disability Ethics Committees (HDEC) (University of Auckland, New Zealand). The original ethics approvals allowed for secondary analyses based on the database created during PREVIEW and also for subgroup studies at each site, which were subject to separate ethics approvals.

Randomization and masking

Glycosylated hemoglobin (HbA_{1c}) was not used to define prediabetes because it was not recommended by World Health Organization or American Diabetes Association as a criterion for diabetes or prediabetes when we drafted and submitted the protocol to the Human Ethics Committee at each intervention centre for approval[1,2]. In addition, in

consideration of the comparability of previous diabetes prevention studies (e.g., the US Diabetes Prevention Program[3] and the Finnish Diabetes Prevention Study[4]), we did not use HbA_{1c}.

At the beginning of the rapid weight loss phase, participants were randomly assigned in a 1:1 ratio to one of the two diet groups, and those in each diet group were randomised (1:1) into one of the two physical activity (PA) groups. The randomization was stratified by sex (females/males) and age group (25–45, 46–54, and 55–70 years) at each centre. Participants were enrolled by study staff. An automated web-response central randomisation system was used to generate random sequences and randomise participants and this system was used to achieve allocation concealment. The allocation order was concealed from staff and investigators and not disclosed to participants until the beginning of the weight maintenance phase. Study investigators, staff, and participants were not blinded because of the nature of the intervention, while all staff involved in statistical analyses were blinded to the randomisation.

Interventions

At the beginning of phase 2, each participant was randomised into one of the four lifestyle intervention groups: 1) a high-protein, low-glycaemic index (GI) diet combined with high intensity PA; 2) a high-protein, low-GI diet combined with moderate intensity PA; 3) a moderate-protein, moderate-GI diet combined with high intensity PA; 4) a moderate-protein, moderate-GI diet combined with moderate intensity PA. The two PA programs included high intensity PA (≥6.0 metabolic equivalents of task for 75 min/week) and moderate intensity PA (3.0–5.9 metabolic equivalents of task for 150 min/week), with a similar target for energy expenditure of more than 4.2 MJ/week (1000 kcal/week).

GI, a numerical system ranging from 0 to 100, is used to measure how quickly carbohydrates in foods raise blood glucose levels. Foods with a high GI (≥70) are considered rapidly digested and absorbed, resulting in a swift spike in blood glucose. A GI

greater than 55 but lower than 70 indicates that a food will likely cause a moderate rise in blood glucose levels after consumption, whereas a GI<55 is considered to be foods that raise blood glucose levels slowly.

All questionnaires, case report forms, booklets, and other materials used in PREVIEW were prepared in English, then translated into the local language using authorised translators at each centre. Before the trial start, standard operating procedures were developed and all study staff were trained. Instruction material for lifestyle changes were also created. In particular, each dietary group was provided with daily eating plans, food-exchange list, and cookbooks to reflect the required macronutrient intakes. The diet and PA recommendations were harmonised across intervention centres using the PREVIEW Behavior Modification Intervention Toolbox. To improve diet and PA compliance, 17 group counselling visits led by instructors were performed throughout the study.

Outcomes

The formulas used to calculate A body shape index (ABSI), visceral adiposity index (VAI), and lipid accumulation product (LAP) were as following: ABSI was calculated as waist circumference/(body mass index, $BMI^{2/3}\times height^{1/2}$)[5]. VAI was calculated as [waist circumference/(39.68+1.88×BMI)]×(triglyceride/1.03)×(1.31/high density lipoprotein-cholesterol) for males and [waist circumference/(36.58+1.89×BMI)]×(triglyceride/0.81)×(1.52/high density lipoprotein-cholesterol) for females[6]. Lipid accumulation product (LAP) was calculated as (waist circumference-65)×triglyceride for males and (waist circumference-58)×triglyceride for females[7].

Dietary intake was assessed using self-reported 4-day food records including three working days and one weekend day. At beginning of the study (phase 1), participants were instructed how to use scales and conventional household measurements, and to record the foods and diets consumed in detail (e.g., type of foods and cooking methods). Food records were collected and unclear cases were discussed and when possible, checked with the

participant to clarify any ambiguities. All data from food records were entered into national nutrient analysis software i.e., Dankost Pro (Denmark), AivoDiet (Finland), Mijn Eetmeter (the Netherlands), Nutritics (the UK), Dial (Spain), Nutrition Calculation (Bulgaria), and Foodworks (Australia and New Zealand) for food and nutrient calculation. The GI and glycaemic load (GL) of each food was obtained using GI databases and for mixed meals the weighted mean GI and GL of the components was used[8].Total GI and GL were calculated according to van Woudenbergh et al.[9]:

$$\mathrm{GI} = \frac{\sum_{i=1}^{n} (\mathrm{GI_i} \times \mathrm{carbohydrates_i})}{\sum_{i=1}^{n} (\mathrm{carbohydrates_i})}$$

$$\mathrm{GL} = \frac{\sum_{i=1}^{n} (\mathrm{GI_i} \times \mathrm{carbohydrates_i})}{100}$$

PA was determined using a 7-day accelerometry (ActiSleep+; ActiGraph LLC, Pensacola, FL). Total PA was expressed by mean activity counts during valid wear time (count/min). Troiano cut points were used to determine time (min/day) spent at different intensity PA categories (sedentary<100, moderate<2020, and vigorous<5999 count/min). Dietary and PA data collected at baseline, 8 weeks, 1 year, and 3 years were used for analysis. According to the PREVIEW main paper, there were no difference between the PA groups within each dietary arm[10].

The outcomes were measured according to prespecified standard operating procedure. Briefly, blood samples were drawn from the participant's antecubital vein during an oral glucose tolerance test. The samples were initially stored at −80°C at each centre and then were transported to the Finnish Institute for Health and Welfare and were centrally analysed. The laboratory (T077) is certified by the Finnish Accreditation Service and complies with the SFS-EN ISO/IEC 17025:2005 standard. Laboratory analyses were conducted using an Architect ci8200 integrated system (Abbott Laboratories, Abbott Park, Illinois). Additional outcomes included changes in body weight, measured under fasting conditions with an empty bladder while wearing only underwear or light clothing. Two measurements were recorded to the nearest 0.1 kg, and the average was calculated. For height assessment,

participants removed their shoes and stood with their heels, buttocks, and upper back against a wall-mounted stadiometer. Height was recorded to the nearest 0.5 cm, with the average of two measurements used for analysis. Waist and hip thigh circumferences were measured to the nearest 0.5 cm using a non-stretch tape while the participant stood upright. Two readings were taken for each site, and the mean value was calculated. Waist circumference was assessed at the midpoint between the lower edge of the rib cage (last floating rib) and the iliac crest at the end of a normal exhalation. Hip circumference was measured at the maximal protrusion between the hips and buttocks, using the same protocol as for waist measurements. Mid-thigh circumference was assessed on the right thigh, with the tape placed horizontally midway between the inguinal crease and the proximal edge of the patella. Body composition including fat mass, fat-free mass, bone mineral content, and bone mineral density was determined by dual energy X-ray absorptiometry in the UK, Australia, and New Zealand and by bioelectrical impedance in Finland and Bulgaria. Fertile women underwent pregnancy testing prior to dual energy x-ray absorptiometry scans.

Statistical analysis

For the primary outcome of the current analysis (i.e., prediabetes remission at 1 and 3 years), we conducted several sensitivity analyses: 1) by imputing missing data using multiple imputation with a fully conditional specification model (Markov chain Monte Carlo). We generated 10 multiple imputed datasets and pooled estimates were reported; 2) by defining prediabetes remission using fasting and 2-h glucose and HbA_{1c} and removing 23 participants who had diabetes based on HbA_{1c} cut points at baseline; 3) by using ADA weight loss target (≥7% of body weight) for diabetes prevention; 4) by further adjusting for PA group; 5) by further adjusting for energy intake at 1 or 3 years; 6) in participants who completed the study. We also conducted auxiliary analyses to compare diabetes incidence between the diet groups using the ITT dataset and Cox proportional hazards models adjusted for age, sex, and centre. Moreover, we explored the associations between prediabetes remission at 1 year and subsequent type 2 diabetes risk (results shown in ESM).

ESM Results

Prediabetes remission

For the auxiliary analyses, 61 cases occurred (31 in the moderate-protein, moderate-GI group and 30 in the high-protein, low-GI group) during the study. There were no differences in 3-year diabetes incidence between the diet groups [5.4% vs 5.1%, hazard ratio, RR 0.97 (0.76, 1.25), *p*=0.831].

The role of body weight and composition in prediabetes remission

Changes in body weight, BMI, waist-to-hip ratio (WHR), fat mass, and HOMA-IR were similar between the two diet groups at 1 year, apart from HOMA-B (a measure of beta cell function) (**ESM Table 5**), where greater decline occurred in the high protein-low GI group. The two diet groups had similar changes at 3 years. Both diet groups had similar changes in 1-year body weight, BMI, WHR change, fat mass and HOMA-IR, but the high protein-low GI group had greater decline in HOMA-B (**ESM Table 5**). The two diet groups had similar changes at 3 years. Within the high-protein, low-GI group, participants who achieved remission at 12 months and 3 years had greater reductions in body weight, BMI, fat mass, HOMA-IR, and HOMA-B but not in waist to hip ratio (**ESM Table 6**). Within the moderate-protein, moderate-GI group, participants who achieved remission at 12 months and 3 years had greater improvements in body weight, BMI, fat mass, and HOMA-IR, but changes in waist to hip ratio and HOMA-B were similar (**ESM Table 6**).

There was no significant interaction between the diet groups and maintenance of ≥8% or ≥7% weight loss at 1 year [≥8% weight loss: RR for interaction 0.90 (95% CI 0.54, 1.51), *p* for interaction=0.689; ≥7% weight loss: RR for interaction 0.76 (0.44, 1.33), *p* for interaction=0.334], whereas at 3 years the effects of the moderate-protein, moderate-GI group on remission of prediabetes were stronger in individuals who maintained 8% or 7% weight loss [≥8% weight loss: RR for interaction 1.93 (1.13, 3.31), *p* for interaction=0.016; ≥7% weight loss: RR for interaction 1.66 (1.07, 2.57), *p* for interaction=0.024] (**ESM Table 7**).

Association between prediabetes remission and subsequent type 2 diabetes risk

Compared with those who did not achieve remission at 1 year, participants who achieved prediabetes remission were not significantly associated with the type 2 diabetes risk at 3 years (RR [95% CI] 0.41 [0.11, 1.56], p=0.159).

We also analyzed the association between prediabetes remission at 1 year and risk of subsequent type 2 diabetes (e.g., diagnosed type 2 diabetes at 2 or 3 years). Compared with non-remission, prediabetes remission at 1 year was significantly associated with a lower risk of type 2 diabetes later (RR [95% CI] 0.18 [0.06, 0.54], p=0.008).

References

1. World Health Organization. Use of glycated haemoglobin (HbA1c) in the diagnosis of diabetes mellitus abbreviated report of a WHO consultation. Use of Glycated Haemoglobin (HbA1c) in the Diagnosis of Diabetes Mellitus Abbreviated Report of a WHO Consultation; 2011: 25.
2. American Diabetes Association. Diagnosis and classification of diabetes mellitus. *Diabetes Care* 2010; **33 Suppl 1**(Suppl 1): S62-9.
3. Knowler WC, Barrett-Connor E, Fowler SE, et al. Reduction in the incidence of type 2 diabetes with lifestyle intervention or metformin. *N Engl J Med* 2002; **346**(6): 393-403.
4. Lindstrom J, Ilanne-Parikka P, Peltonen M, et al. Sustained reduction in the incidence of type 2 diabetes by lifestyle intervention: follow-up of the Finnish Diabetes Prevention Study. *Lancet* 2006; **368**(9548): 1673-9.
5. Krakauer NY, Krakauer JC. A new body shape index predicts mortality hazard independently of body mass index. *PLoS One* 2012; **7**(7): e39504.
6. Amato MC, Giordano C, Galia M, et al. Visceral Adiposity Index: a reliable indicator of visceral fat function associated with cardiometabolic risk. *Diabetes Care* 2010; **33**(4): 920-2.
7. Chen J, Sun H, Qiu S, Tao H, Yu J, Sun Z. Lipid Accumulation Product Combined With Urine Glucose Excretion Improves the Efficiency of Diabetes Screening in Chinese Adults. *Front Endocrinol (Lausanne)* 2021; **12**: 691849.
8. Wolever Thomas MS, Ming Y, Yi ZX, Fiona A, Brand-Miller JC. Food glycemic index, as given in Glycemic Index tables, is a significant determinant of glycemic responses elicited by composite breakfast meals. *Am J Clin Nutr*; **83**(6): 1306-12.
9. Van WGJ, Anneleen K, Sijbrands EJG, Albert H, Witteman JCM, Feskens EJM. Glycemic Index and Glycemic Load and Their Association with C-Reactive Protein and Incident Type 2 Diabetes. *J Nutr Metab* 2011; **2011**(3): 623076.
10. Raben A, Vestentoft PS, Brand-Miller J, et al. The PREVIEW intervention study: Results from a 3-year randomized 2 x 2 factorial multinational trial investigating the role of protein, glycaemic index and physical activity for prevention of type 2 diabetes. *Diabetes Obes Metab* 2021; **23**(2): 324-37.

ESM Table 1. Characteristics of completers and non-completers

	Total population			HP-LGI participants			MP-MGI participants		
	Completers (*N*=962)	Non-completers (*N*=894)	*p* value	Completers (*N*=497)	Non-completers (*N*=454)	*p* value	Completers (*N*=483)	Non-completers (*N*=440)	*p* value
Age range, years	25–70	25–70	-			-			-
Age, years	57 (47, 63)	50 (40, 60)	<0.001	58 (47, 63)	49 (40, 59)	<0.001	56 (46, 63)	51 (41, 60)	<0.001
Age group			<0.001			<0.001			<0.001
25–45 years	224 (23.3)	365 (40.8)	-	106 (22.1)	196 (43.2)	-	118 (24.4)	169 (38.4)	-
46–54 years	151 (15.7)	140 (15.7)	-	73 (15.2)	70 (15.4)	-	78 (16.1)	70 (15.9)	-
55–70 years	587 (61.0)	389 (43.5)	-	300 (62.6)	188 (41.4)	-	287 (59.4)	201 (45.7)	-
Sex			0.048			0.040			0.459
Females	619 (64.3)	614 (68.7)	-	303 (63.3)	316 (69.6)	-	316 (65.4)	298 (67.7)	-
Males	280 (29.1)	343 (31.3)	-	176 (36.7)	138 (30.4)	-	167 (34.6)	142 (32.3)	-
Race and ethnicity			<0.001			<0.001			0.020
White	891 (92.6)	772 (86.4)	-	454 (94.8)	389 (85.7)	-	437 (90.5)	383 (87.0)	-
Other[a]	32 (7.4)	122 (13.6)	-	43 (5.2)	65 (14.3)	-	46 (9.5)	57 (13.0)	-
Body weight, kg	92.8 (82.7, 104.7)	101.4 (88.4, 116.3)	<0.001	92.8 (82.5, 104.4)	102.4 (89.4, 118.3)	<0.001	92.7 (83.3, 105.4)	100.5 (87.9, 114.3)	<0.001
Height, m	1.68 (1.62, 1.76)	1.67 (1.61, 1.74)	0.038	1.68 (1.62, 1.75)	1.66 (1.61, 1.73)	<0.001	1.67 (1.62, 1.76)	1.67 (1.61, 1.74)	<0.001
BMI, kg/m^2	32.6 (29.9, 36.2)	35.6 (32.1, 40.5)	<0.001	32.1 (29.9, 36.2)	36.1 (32.3, 41.4)	<0.001	32.7 (29.9, 36.2)	35.3 (31.9, 39.5)	<0.001
Weight status			<0.001			<0.001			<0.001
Overweight[b]	249 (25.9)	109 (12.2)	-	126 (26.3)	47 (10.4)	-	123 (25.5)	62 (14.1)	-
Obesity[c]	713 (74.1)	785 (87.8)	-	353 (73.7)	407 (89.6)	-	360 (74.5)	378 (85.9)	-
Fat mass, kg	38.0 (31.5, 46.2)	43.7 (35.7, 54.1)	<0.001	37.5 (31.2, 45.4)	43.5 (36.0, 54.6)	<0.001	38.8 (31.9, 46.9)	43.9 (35.6, 52.6)	<0.001
Fat mass index, kg/m^2	13.5 (11.0, 16.4)	15.6 (13.0, 19.3)	<0.001	13.1 (11.0, 16.2)	15.8 (13.2, 19.9)	<0.001	13.7 (11.1, 16.4)	15.5 (12.9, 19.1)	<0.001
Fat-free mass, kg	52.6 (47.0, 63.2)	55.2 (48.4, 65.3)	<0.001	52.6 (47.4, 62.8)	55.8 (48.4, 65.6)	0.003	52.5 (46.8, 63.7)	55.0 (48.4, 64.9)	0.017

Fat-free mass index, kg/m^2	19.1 (17.5, 20.8)	20.1 (18.3, 21.9)	<0.001	19.1 (17.6, 20.9)	20.3 (18.3, 22.1)	<0.001	19.0 (17.4, 20.8)	19.9 (18.2, 21.7)	<0.001
Waist circumference, cm	107.3 (99.0, 116.0)	111.5 (102.0, 121.4)	<0.001	107.25 (100.25, 115.75)	112.0 (102.5, 123.4)	<0.001	107.3 (98.5, 116.8)	111.0 (101.8, 120.0)	<0.001
Hip circumference, cm	113.0 (107.0, 121.0)	118.8 (111.3, 130.0)	<0.001	112.3 (106.8, 120.8)	119.00 (112.0, 130.5)	<0.001	113.9 (107.5, 121.5)	118.1 (110.3, 128.9)	<0.001
WHR	0.94 (0.88, 1.00)	0.92 (0.87, 0.99)	0.036	0.94 (0.88, 1.01)	0.92 (0.87, 0.99)	0.023	0.93 (0.87, 1.00)	0.92 (0.87, 0.99)	0.481
ABSI	0.081 (0.077, 0.084)	0.079 (0.075, 0.083)	<0.001	0.08 (0.08, 0.08)	0.08 (0.07, 0.08)	<0.001	0.08 (0.08, 0.08)	0.08 (0.08, 0.08)	0.003
VAI	1.80 (1.25, 2.61)	1.89 (1.35, 2.83)	0.004	1.81 (1.24, 2.56)	2.01 (1.37, 2.94)	0.020	1.80 (1.26, 2.65)	1.83 (1.35, 2.67)	0.083
LAP	61.1 (42.5, 86.4)	70.5 (47.2, 102.9)	<0.001	61.2 (42.5, 85.3)	72.3 (47.5, 106.8)	<0.001	60.8 (42.5, 88.9)	68.0 (47.0, 98.6)	0.001
Fasting plasma glucose, mmol/l	6.1±0.7	6.2±0.8	0.603	6.2±0.7	6.2±0.7	0.348	6.2±0.6	6.2±0.8	0.151
2 h plasma glucose, mmol/l	7.6±2.2	7.8±2.2	0.061	7.6±2.2	7.8±2.2	0.097	7.5±2.1	7.7±2.2	0.255
HbA_{1c}, mmol/mol	36.7±3.6	36.6±4.1	0.343	5.5±0.3	5.5±0.4	0.500	5.5±0.4	5.5±0.4	0.934
HbA_{1c}, %	5.5±0.3	5.5±0.4	0.343	36.7±3.3	36.5±4.0	0.447	36.7±3.8	36.7±4.2	0.897

Values are mean±SD or median (25th–75th percentiles) for continuous variables and *n* (%) for categorical variables

[a]Including Asian, Black, Arabic, Hispanic, and other

[b]Overweight is defined as a BMI≥25 and <30 kg/m^2

[c]Obesity is defined as a BMI≥30 kg/m^2

HP-LGI, high-protein, low glycaemic index diet; MP-MGI, moderate-protein, moderate-glycaemic index diet

ESM Table 2. Dietary intake between the two diet group over 156 weeks

	Diet group	Population	Baseline	1 years	3 years	Model 1			Model 2		
						p for group and time interaction	*p* for diet group	*p* for time	*p* for group and time interaction	*p* for diet group	*p* for time
Carbohydrate, E%	HP-LGI	Total population	40.1 (0.3)	37.9 (0.4)*	37.8 (0.4)*	<0.001	-	-	<0.001	-	-
	MP-MGI	Total population	40.1 (0.3)	43.3 (0.4)	41.3 (0.4)						
Glycaemic index	HP-LGI	Total population	56.0 (0.3)	51.0 (0.4)*	51.6 (0.5)*	<0.001	-	-	<0.001	-	-
	MP-MGI	Total population	56.1 (0.3)	54.6 (0.4)	54.3 (0.4)						
Glycaemic load	HP-LGI	Total population	118.9 (1.8)	83.4 (1.8)*	80.8 (1.9)*	<0.001	-	-	<0.001	-	-
	MP-MGI	Total population	117.9 (1.7)	95.4 (1.7)	90.6 (1.8)						
Protein, E%	HP-LGI	Total population	17.9 (0.1)	22.1 (0.2)*	21.2 (0.3)*	<0.001	-	-	<0.001	-	-
	MP-MGI	Total population	17.7 (0.1)	18.4 (0.2)	18.8 (0.3)						
Fat, E%	HP-LGI	Total population	36.7 (0.3)	34.9 (0.4)	35.4 (0.4)	0.054	0.476	<0.001	0.055	0.686	<0.001
	MP-MGI	Total population	37.3 (0.3)	33.1 (0.3)	34.8 (0.4)						
Dietary fibre, g/day	HP-LGI	Total population	22.4 (0.4)	23.0 (0.4)	21.0 (0.4)	0.051	0.269	<0.001	0.060	0.114	<0.001
	MP-MGI	Total population	21.6 (0.3)	22.4 (0.4)	21.6 (0.4)						
Energy, kJ/day	HP-LGI	Total population	8865.7 (108.6)	7191.2 (108.1)	6887.1 (110.4)	0.056	0.054	<0.001	-	-	-
	MP-MGI	Total population	8749.9 (104.7)	6762.7 (105.9)	6771.2 (106.4)						
Energy, kcal/day	HP-LGI	Total population	2110.9 (25.8)	1712.2 (25.7)	1639.8 (26.3)	0.057	0.054	<0.001	-	-	-
	MP-MGI	Total population	2083.3 (24.9)	1610.2 (25.2)	1612.2 (25.3)						
	HP-LGI	Females	2072.4 (42.9)	1710.8 (42.7)	1623.4 (43.3)	0.351	0.054	<0.001	-	-	-
	HP-LGI	Males	2129.8 (30.6)	1712.4 (30.3)	1647.7 (31.2)						
	MP-MGI	Females	2118.0 (40.7)	1647.2 (41.3)	1596.2 (40.3)						
	MP-MGI	Males	2064.5 (29.7)	1591.0 (29.9)	1622.4 (30.6)						
	HP-LGI	Younger people	2129.8 (30.6)	1712.4 (30.3)	1647.7 (31.2)	0.231	0.054	<0.001	-	-	-

HP-LGI	Middle-aged people	2072.4 (42.9)	1710.8 (42.7)	1623.4 (43.3)						
HP-LGI	Older people	2064.5 (29.7)	1591.0 (29.9)	1622.4 (30.6)						
MP-MGI	Younger people	2118.0 (40.7)	1647.2 (41.3)	1596.2 (40.3)						
MP-MGI	Middle-aged people	2089.5 (56.4)	1517.9 (60.7)	1605.3 (61.4)						
MP-MGI	Older people	2052.7 (31.8)	1624.9 (31.3)	1602.2 (31.4)						
HP-LGI	High intensity physical activity	2128.7 (35.9)	1689.5 (35.3)	1601.9 (36.5)						
HP-LGI	Moderate intensity physical activity	2094.6 (35.1)	1735.9 (35.3)	1677.0 (35.8)	0.081	0.054	<0.001	-	-	-
MP-MGI	High intensity physical activity	2088.2 (34.2)	1588.2 (34.6)	1593.5 (34.8)						
MP-MGI	Moderate intensity physical activity	2079.4 (34.3)	1632.2 (34.5)	1631.2 (34.6)						

Data are estimated marginal mean (standard error). Analyses were performed using linear mixed models. Model 1 was adjusted for sex, age group, and time as fixed effects and participant identifier and intervention centre as random effects. Model 2 was further adjusted for energy intake. Time by diet group interaction terms were added. Post hoc analyses with pairwise comparisons (independent-samples *t* test) were performed to compare dietary intake at each time point, where appropriate

*Significant differences between the two diet groups. HP-LGI, high-protein, low glycaemic index diet; MP-MGI, moderate-protein, moderate-glycaemic index diet

ESM Table 3. Physical activity between the two diet group over 3 years

	Diet group	Baseline	1 years	3 years	*p* for group and time interaction	*p* for group	*p* for time
Sedentary time, min/day	HP-LGI	509.3 (3.6)	510.7 (6.7)	588.4 (4.9)	0.469	0.839	<0.001
	MP-MGI	586.9 (3.6)	517.2 (6.8)	591.5 (4.9)			
Light physical activity, min/day	HP-LGI	314.6 (3.1)	280.6 (5.1)	297.4 (4.1)	0.437	0.727	<0.001
	MP-MGI	315.6 (3.0)	276.1 (5.1)	302.5 (4.1)			
Moderate physical activity, min/day	HP-LGI	28.4 (0.7)	29.3 (1.1)	31.1 (1.2)	0.844	0.491	0.016
	MP-MGI	28.9 (0.7)	30.5 (1.1)	31.2 (1.2)			
Vigorous physical activity, min/day	HP-LGI	0.4 (0.09)	1.4 (0.3)	1.1 (0.2)	0.791	0.748	<0.001
	MP-MGI	0.5 (0.09)	1.3 (0.3)	1.1 (0.2)			
Moderate-to-vigorous physical activity, min/day	HP-LGI	28.8 (0.8)	30.7 (1.2)	32.2 (1.3)	0.883	0.510	0.001
	MP-MGI	29.4 (0.7)	31.8 (1.2)	32.2 (1.2)			
Total physical activity, count/min	HP-LGI	296.3 (4.3)	320.8 (5.5)	302.4 (6.2)	0.882	0.457	<0.001
	MP-MGI	299.3 (4.2)	327.3 (5.6)	306.1 (6.1)			

Data are estimated marginal mean (standard error). Analyses were performed using linear mixed models. Models were adjusted for sex, age group, and time as fixed effects and participant identifier and intervention centre as random effects. Time by diet group interaction terms were added. HP-LGI, high-protein, low glycaemic index diet; MP-MGI, moderate-protein, moderate-glycaemic index diet

ESM Table 4. Changes in body weight and composition measures from baseline between the two diet group over 3 years

	Diet group	Baseline	8 weeks	1 years	3 years	*p* for group and time interaction	*p* for group	*p* for time
Body weight	HP-LGI	104.4 (0.9)	93.1 (0.9)	95.5 (0.9)	99.7 (0.9)	0.887	0.805	<0.001
	MP-MGI	104.2 (0.9)	93.0 (0.9)	95.2 (0.9)	99.4 (0.9)			
Percentage of weight loss	HP-LGI	-0.7 (0.2)	-12.1 (0.2)	-9.8 (0.2)	-5.5 (0.2)	0.700	0.540	<0.001
	MP-MGI	-0.7 (0.2)	-12.0 (0.2)	-10.0 (0.2)	-5.6 (0.2)			
BMI	HP-LGI	33.6 (0.3)	29.7 (0.3)	30.5 (0.3)	32.0 (0.3)	0.849	0.398	<0.001
	MP-MGI	33.4 (0.3)	29.5 (0.3)	30.2 (0.3)	31.7 (0.3)			
Fat mass	HP-LGI	37.7 (0.7)	29.5 (0.7)	30.3 (0.7)	34.5 (0.7)	0.905	0.623	<0.001
	MP-MGI	37.9 (0.7)	29.7 (0.7)	30.5 (0.7)	35.0 (0.7)			
Fat mass index	HP-LGI	12.2 (0.2)	9.3 (0.2)	9.6 (0.2)	11.1 (0.2)	0.877	0.964	<0.001
	MP-MGI	12.2 (0.2)	9.3 (0.2)	9.6 (0.2)	11.2 (0.2)			
Fat-free mass	HP-LGI	66.5 (0.4)	63.7 (0.4)	65.0 (0.4)	64.9 (0.4)	0.917	0.527	<0.001
	MP-MGI	66.3 (0.4)	63.5 (0.4)	64.7 (0.4)	64.7 (0.4)			
Fat-free mass index	HP-LGI	21.3 (0.1)	20.3 (0.1)	20.8 (0.1)	20.7 (0.1)	0.904	0.143	<0.001
	MP-MGI	21.2 (0.1)	20.2 (0.1)	20.6 (0.1)	20.6 (0.1)			
WHR	HP-LGI	1.027 (0.003)	0.997 (0.003)	1.003 (0.003)	1.014 (0.004)	0.058	0.476	<0.001
	MP-MGI	1.024 (0.003)	0.997 (0.003)	0.997 (0.004)	1.015 (0.004)			
ABSI	HP-LGI	0.084 (0.0002)	0.083 (0.0002)	0.083 (0.0002)	0.084 (0.0003)	0.008	-	-
	MP-MGI	0.084 (0.0002)	0.083 (0.0002)	0.083 (0.0002)	0.084 (0.0003)			
VAI	HP-LGI	2.03 (0.06)	1.53 (0.06)	1.57 (0.06)	1.68 (0.07)	0.461	0.665	<0.001
	MP-MGI	2.03 (0.06)	1.51 (0.06)	1.61 (0.06)	1.77 (0.07)			
LAP	HP-LGI	80.4 (1.9)	48.6 (1.9)	59.5 (2.0)	66.0 (2.1)	0.360	0.769	<0.001
	MP-MGI	79.3 (1.9)	48.4 (1.9)	60.2 (2.0)	68.4 (2.1)			

Data are estimated marginal mean (standard error). Analyses were performed using a linear mixed model adjusted for sex, age group, and time as fixed effects and participant identifier and intervention centre as random effects. Time by diet group interaction terms were added. *Post hoc* analyses with pairwise comparisons (independent-samples *t* test) were performed to compare dietary intake at each time point, where appropriate

*Significant differences between the two diet groups. HP-LGI, high-protein, low glycaemic index diet; MP-MGI, moderate-protein, moderate-glycaemic index diet

ESM Table 5. Changes in body weight and composition, insulin resistance, and beta cell function across intervention groups

	HP-LGI	MP-MGI	*p* value
At 1 year			
Change in weight from baseline, kg	-9.0±6.6	-9.1±6.9	0.703
Change in BMI from baseline, kg/m^2	-3.1±2.3	-3.2±2.4	0.640
Change in WHR from baseline	-0.02±0.05	-0.03±0.06	0.285
Change in fat mass from baseline, kg	-7.4±5.9	-7.5±5.9	0.883
Change in HOMA-IR from baseline	-1.2±2.2	-1.0±1.6	0.135
Change in HOMA-B from baseline	-22.5±43.5	-15.5±61.8	0.023
At 3 years			
Change in weight from baseline, kg	-4.7±7.1	-4.8±7.5	0.853
Change in BMI from baseline, kg/m^2	-1.6±2.4	-1.7±2.6	0.851
Change in WHR from baseline	-0.01±0.05	-0.009±0.06	0.085
Change in fat mass from baseline, kg	-3.2±5.8	-3.0±7.1	0.554
Change in HOMA-IR from baseline	-0.8±2.5	-0.5±1.8	0.061
Change in HOMA-B from baseline	-16.6±47.9	-12.9±50.8	0.256

Data are mean±standard deviation. HP-LGI, high-protein, low glycaemic index diet; MP-MGI, moderate-protein, moderate-glycaemic index diet

ESM Table 6. Changes in body compositions, insulin resistance, and beta cell function between participants who achieved and did not achieve remission in each diet group

	HP-LGI			MP-MGI		
	Remission	Non-remission	*p* value	Remission	Non-remission	*p* value
At 1 year						
Change in weight from baseline, kg	-11.8±7.3	-8.2±6.2	<0.001	-11.3±7.9	-8.3±6.4	<0.001
Change in BMI from baseline, kg/m^2	-4.1±2.6	-2.9±2.2	<0.001	-3.9±2.8	-2.9±2.2	<0.001
Change in WHR from baseline	-0.03±0.06	-0.02±0.05	0.230	-0.02±0.06	-0.03±0.05	0.092
Change in fat mass from baseline, kg	-9.8±6.1	-6.8±5.7	<0.001	-9.4±6.7	-6.8±5.5	<0.001
Change in HOMA-IR from baseline	-1.6±1.6	-1.1±2.3	0.004	-1.3±1.4	-0.9±1.7	0.014
Change in HOMA-B from baseline	-32.7±55.3	-19.8±39.5	0.013	-14.0±101.4	-16.1±39.3	0.807
At 3 years						
Change in weight from baseline, kg	-6.3±7.4	-4.5±7.0	0.057	-7.9±8.6	-4.2±7.1	0.000
Change in BMI from baseline, kg/m^2	-2.2±2.5	-1.5±2.4	0.034	-2.8±3.0	-1.4±2.4	<0.001
Change in WHR from baseline	-0.03±0.06	-0.01±0.05	0.010	-0.005±0.06	-0.009±0.05	0.523
Change in fat mass from baseline, kg	-4.9±6.1	-2.9±5.7	0.010	-6.2±7.1	-2.2±7.0	<0.001
Change in HOMA-IR from baseline	-1.2±3.1	-0.7±2.4	0.191	-1.0±1.4	-0.4±1.9	<0.001
Change in HOMA-B from baseline	-17.1±49.6	-17.9±45.4	0.893	-20.8±78.1	-11.3±40.8	0.256

Data are mean±standard deviation. HP-LGI, high-protein, low glycaemic index diet; MP-MGI, moderate-protein, moderate-glycaemic index diet

ESM Table 7. Associations between dietary patterns and prediabetes remission among participants with different weight loss status at 3 years

	Risk ratio (95% CI)	*p*-value	*p* for interaction
Among participants with weight loss ≥7%			
HP-LGI	Reference		
MP-MGI	1.79 (1.24-2.59)	0.008	0.016
Among participants with weight loss <7%			
HP-LGI	Reference		
MP-MGI	0.88 (0.61-1.26)	0.416	
Among participants with weight loss ≥8%			
HP-LGI	Reference		
MP-MGI	1.71 (1.23-2.39)	0.007	0.024
Among participants with weight loss <8%			
HP-LGI	Reference		
MP-MGI	0.98 (0.74-1.32)	0.901	

Analyses were performed using modified Poisson regression models. Models were adjusted for sex (males/females) and age group (25–45, 46–54, and 55–70 years of age) as fixed effects and intervention centre as a random effect. The interaction between dietary patterns and prediabetes remission was calculated by adding interaction term of diet group with maintenance of weight loss target in above models. HP-LGI, high-protein, low glycaemic index diet; MP-MGI, moderate-protein, moderate-glycaemic index diet

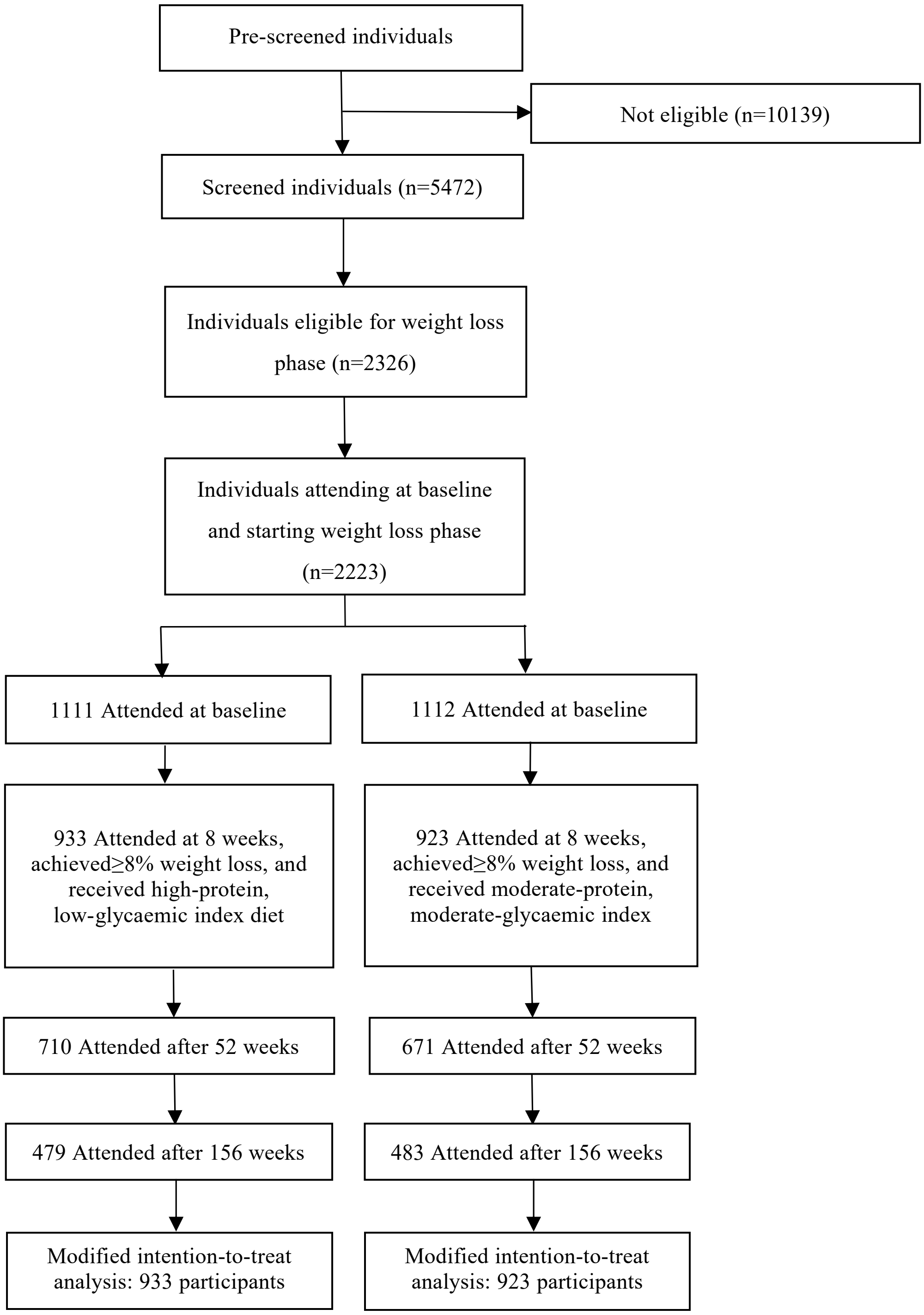

ESM Fig. 1 Trial flow diagram.

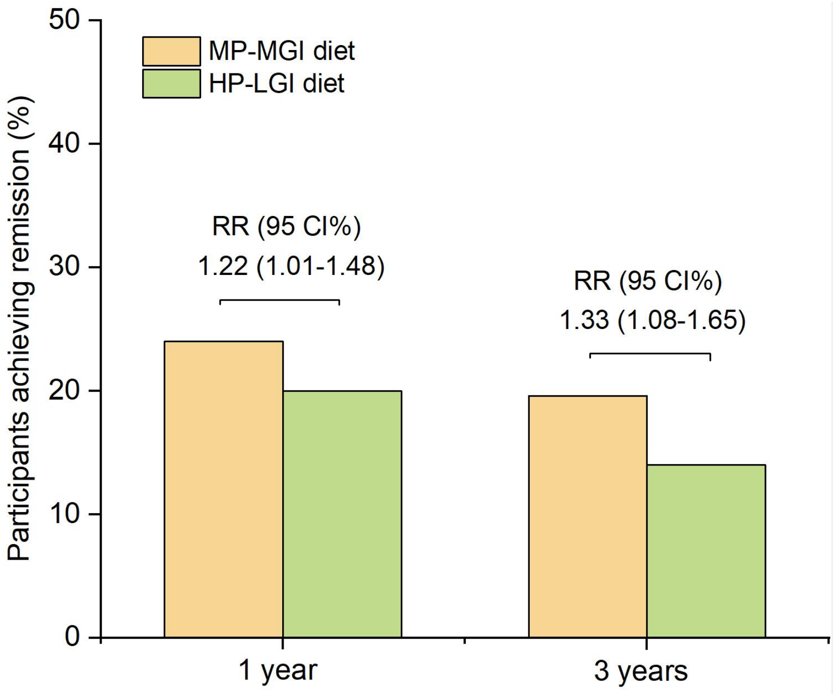

ESM Fig. 2 Proportion of participants who achieved prediabetes remission in each diet group over 3 years. Prediabetes remission was defined using fasting plasma glucose, 2 h fasting plasma glucose, and HbA_{1c}. The RR and 95% CI of prediabetes remission at 1 and 3 years between the diet groups were estimated using modified Poisson regression models, adjusting for sex (males/females) and age group (25–45, 46–54, and 55–70 years of age) as fixed effects and intervention centre as a random effect. The data analyses were based on the modified intention-to-treat analyses without missing data imputation. The high-protein, low-glycaemic index diet was considered the reference group. HP-LGI, high-protein, low glycaemic index diet; MP-MGI, moderate-protein, moderate-glycaemic index diet

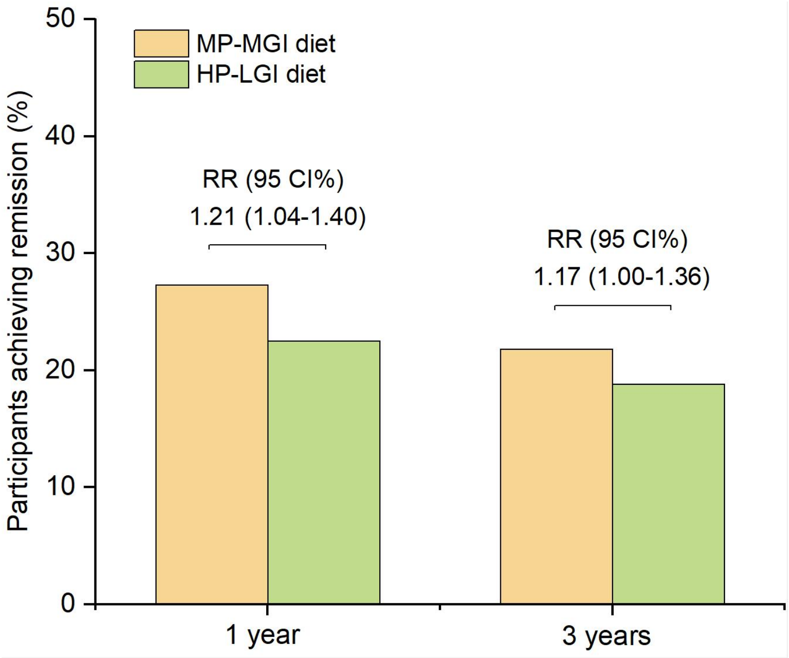

ESM Fig. 3 Proportion of participants who achieved prediabetes remission in each diet group over 3 years. Prediabetes remission was defined using fasting plasma glucose and 2 h fasting plasma glucose. The RR and 95% CI of prediabetes remission at 1 and 3 years between the diet groups were estimated using modified Poisson regression models, adjusting for sex (males/females) and age group (25–45, 46–54, and 55–70 years of age) as fixed effects and intervention centre as a random effect. The data analyses were based on the modified intention-to-treat analyses with missing data imputation. The high-protein, low-glycaemic index diet was considered the reference group. HP-LGI, high-protein, low glycaemic index diet; MP-MGI, moderate-protein, moderate-glycaemic index diet

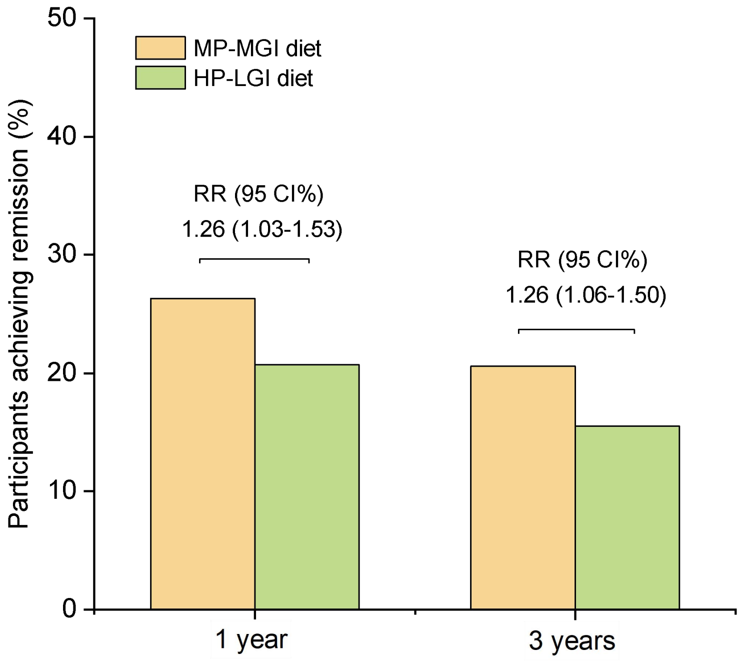

ESM Fig. 4 Proportion of participants who achieved prediabetes remission in each diet group over 3 years. Prediabetes remission was defined using fasting plasma glucose and 2 h fasting plasma glucose. The RR and 95% CI of prediabetes remission at 1 and 3 years between the diet groups were estimated using modified Poisson regression models, adjusting for sex (males/females), age group (25–45, 46–54, and 55–70 years of age), and physical activity group as fixed effects and intervention centre as a random effect. The data analyses were based on the modified intention-to-treat analyses without missing data imputation. The high-protein, low-glycaemic index diet was considered the reference group. HP-LGI, high-protein, low glycaemic index diet; MP-MGI, moderate-protein, moderate-glycaemic index diet

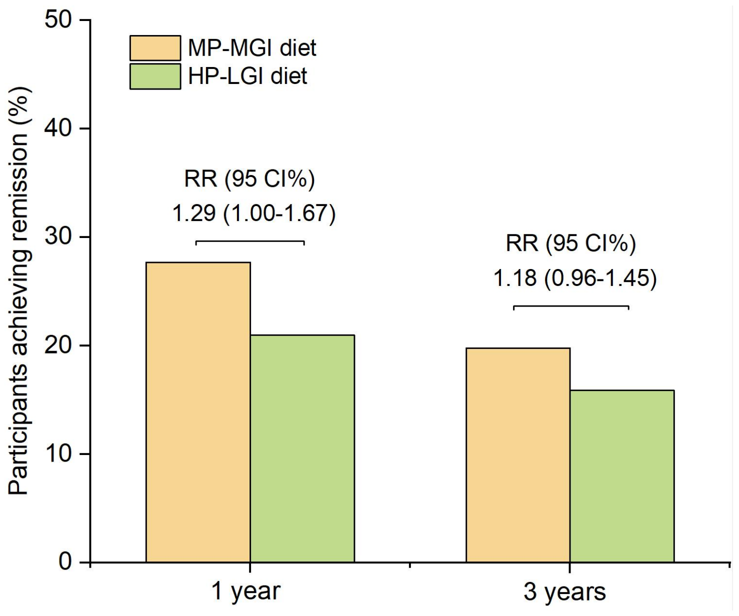

ESM Fig. 5 Proportion of participants who achieved prediabetes remission in each diet group over 3 years. Prediabetes remission was defined using fasting plasma glucose and 2 h fasting plasma glucose. The RR and 95% CI of prediabetes remission at 1 and 3 years between the diet groups were estimated using modified Poisson regression models, adjusting for sex (males/females), age group (25–45, 46–54, and 55–70 years of age), and energy intake at 1 or 3 years as fixed effects and intervention centre as a random effect. The data analyses were based on the modified intention-to-treat analyses without missing data imputation. The high-protein, low-glycaemic index diet was considered the reference group. HP-LGI, high-protein, low glycaemic index diet; MP-MGI, moderate-protein, moderate-glycaemic index diet

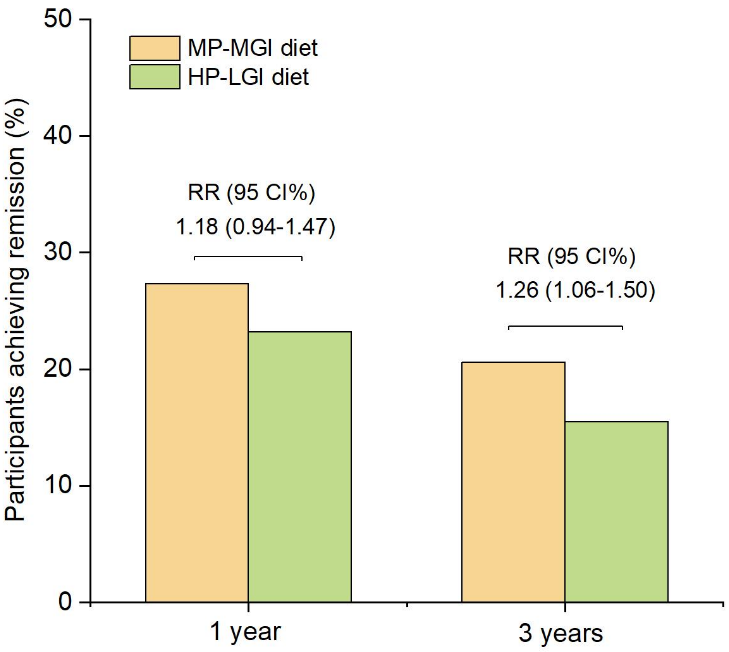

ESM Fig. 6 Proportion of participants who achieved prediabetes remission in each diet group over 3 years in complete-case analyses. Prediabetes remission was defined using fasting plasma glucose and 2 h fasting plasma glucose. RR and 95% CI of prediabetes remission at 1 and 3 years between the diet groups were estimated using modified Poisson regression models, adjusting for sex (males/females) and age group (25–45, 46–54, and 55–70 years of age) as fixed effects and intervention centre as a random effect. The data analyses were based on the complete-case analyses without missing data imputation. The high-protein, low-glycaemic index diet was considered the reference group. HP-LGI, high-protein, low glycaemic index diet; MP-MGI, moderate-protein, moderate-glycaemic index diet

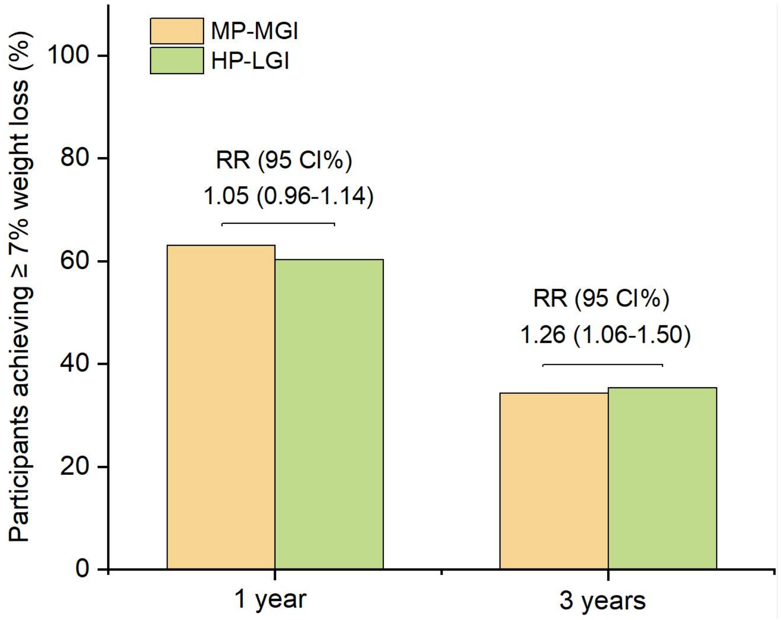

ESM Fig. 7 Proportion of participants who achieved weight loss target (≥7%) according to the American Diabetes Association recommendation in each diet group over 3 years. Prediabetes remission was defined using fasting plasma glucose and 2 h fasting plasma glucose. The RR and 95% CI of prediabetes remission at 1 and 3 years between the diet groups were estimated using modified Poisson regression models, adjusting for sex (males/females) and age group (25–45, 46–54, and 55–70 years of age) as fixed effects and intervention centre as a random effect. The data analyses were based on the modified intention-to-treat analyses without missing data imputation. The high-protein, low-glycaemic index diet was considered the reference group. HP-LGI, high-protein, low glycaemic index diet; MP-MGI, moderate-protein, moderate-glycaemic index diet
